# Quality and accuracy of gastric cancer related videos in social media videos platforms

**DOI:** 10.1186/s12889-022-14417-w

**Published:** 2022-11-05

**Authors:** Ren-hao Hu, Hai-bin Zhang, Biao Yuan, Ke-hui Zhang, Jia-yi Xu, Xi-mao Cui, Tao Du, Chun Song, Shun Zhang, Xiao-hua Jiang

**Affiliations:** 1 Department of Gastrointestinal Surgery, Shanghai East Hospital, Tongji University, Shanghai, China; 2 Center of Digestive Endoscopy, Shanghai East Hospital, Tongji University, Shanghai, China

**Keywords:** Gastric cancer; Social media; Public health; Content analysis

## Abstract

**Background:**

Gastric cancer is a major public health problem worldwide. Social media has affected public’s daily lives in ways no one ever thought possible. Both TikoTok and its Chinese version Douyin are the most popular short video posting platform. This study aimed to evaluate the quality, accuracy, and completeness of videos for gastric cancer on TikTok and Douyin.

**Methods:**

The terms “gastric cancer” was searched on TikTok in both English and Japanese, and on Douyin in Chinese. The first 100 videos in three languages (website’s default setting) were checked. QUality Evaluation Scoring Tool (QUEST) and DISCERN as the instrument for assessing the quality of the information in each video. Content was analysed under six categories (aetiology, anatomy, symptoms, preventions, treatments, and prognosis). The educational value and completeness were evaluated with a checklist developed by the researchers.

**Results:**

A total of 78 videos in English, 63 in Japanese, and 99 in Chinese were analyzed. The types of sources were as follows: 6.4% in English, 4.8% in Japanese, and 57.6% in Chinese for health professionals; 93.6% in English, 95.2% in Japanese, and 3.0% in Chinese for private users; none in English and Japanese, but 39.4% in Chinese for other sources. In all, 20.5% in English, 17.5% in Japanese, and 93.9% in Chinese of videos had useful information about gastric cancer. Among the useful videos, the videos published in Chinese had the highest QUEST(p < 0.05) and DISCERN scores(p < 0.05), followed by those published in Japanese. Among the educational videos, prognosis in English (37.5%), symptoms in Japanese (54.5%), and prevention in Chinese (47.3%) were the most frequently covered topic.

**Conclusions:**

TikTok in English and Japanese might not fully meet the gastric cancer information needs of public, but Douyin in Chinese was the opposite.

## Background

With more than 1 million new cases and 769,000 deaths worldwide, gastric cancer was the fifth most frequent cancer and fourth in cancer-related deaths in 2020 [1]. The incidence of gastric cancer mortality was 37.5% in Japan [2] and 39.9% in the United States [3] compared with 48.6% in China [4].

Social media has affected the public’s daily lives in ways that no one ever thought possible. In July 2021, there were a reported 4.48 billion social media users, equating to more than 57 percent of the total global population [5]. Social media platforms, constituting a powerful means of communication, are increasingly used for health information dissemination. TikTok is one of the most popular social media platforms with more than 1.1 billion monthly active users, 130 million of whom are in the United States [5]. However, TikTok cannot be used in China because of internet censorship. Douyin, the Chinese version of TikTok, has averaged more than 600 million daily active users [6]. Douyin is the most popular short video platform in mainland China. TikTok for global users and Douyin for Chinese users offer the same features and tools.

As the fastest-growing social media applications, their potential as educational tools for health-related content cannot be overlooked. Several studies document health-related topics that can be found on the sites, such as recovering from eating disorders [7], sex education [8] and cancer treatment [9, 10]. However, videos posted on social media are not peer‐reviewed and are commonly ranked according to popularity. Like other social media platforms, the spread of misinformation is a concern on TikTok [11]. Misinformation can confuse the public about diseases and dissuade patients from pursuing treatment. A few studies document the spread of public health-related misinformation on the topics of COVID-19 [12], vaccines [12] and other diseases, such as prostate cancer [13]. A recent study showed that misinformation regarding COVID-19 has undermined public health efforts to control the novel coronavirus [14].

To date, the characteristics of TikTok videos focusing on gastric cancer are unknown. Therefore, this study aims to assess the content, accuracy, and completeness of social media about gastric cancer on TikTok in multiple countries. We also want to share our thoughts on important future directions for managing social media for gastric cancer.

## Materials and methods

We queried the TikTok and Douyin mobile application on August 17, 2021, to locate videos that included any information about gastric cancer. The keyword “gastric cancer” was searched on TikTok in both English and Japanese and on Douyin in Chinese to identify related video clips. The results were sorted according to the applications’ proprietary search algorithm. The first 100 most popular videos were gathered and analyzed. The languages were limited to English and Japanese in TikTok and Chinese in Doyin. Videos that were duplicated, had no sound and were not directly related to gastric cancer were excluded.

Each video was assessed for content quality by two independent gastroenterological surgeons. All coders had studied in Japan at least one year and had sufficient experience in the diagnosis and management of gastric cancer. Any disagreements were discussed until a consensus was reached. The videos were further categorized as useful or useless according to educational content. Useful definitions contained scientifically correct information such as etiology, anatomy, symptoms, prevention, treatment, or prognosis. Useless definitions only addressed personal experience or testimony without any scientific content.

We employed the Quality Evaluation Scoring Tool (QUEST) and DISCERN as the instruments for assessing the quality of the information in each video. The QUEST has been confirmed to be a valid, reliable appraisal tool for websites [15]. It has a scoring matrix and range of possible total scores of 0–28 with a higher score indicating better quality. DISCERN has been one of the most widely adopted instruments for assessing the quality of health information [16]. It consisted of 16 questions in total, with each question scored from 1 to 5 points. Questions were divided into three sections: reliability (questions 1–8), quality information about treatment options (questions 9–15), and overall score (question 16).

To date, there are no validated tools for assessing the video content of gastric cancer. We developed a completeness checklist for evaluating gastric cancer video quality as shown in Table 1. The six categories cover most aspects of gastric cancer from etiology and treatment to prognosis. We first applied the completeness score in our previous study about internet videos and colorectal cancer [17]. The completeness score compared with that of a previous study that contained the same six categories, but the details were slightly different according to the different types of cancer.

**Table 1:** Completeness checklist

Content	Description
Aetiology	Precancerous lesion
	Heredity
	Eating habits
Anatomy	-
Symptoms	Anemia
	Nausea and vomit
	Anorexia
	Early satiety
	Dysphagia
	Weight loss
	Abdominal pain
	Blood in the stool
Preventions	Screening
	Daily habits
Treatments	Surgery
	Chemotherapy
	Radiotherapy
Prognosis	TNM stage
	Perioperative treatments
	Others

Statistical analysis was performed with SPSS Version 23 software. Data were summarized as frequencies (n) and percentages (%) for categorical variables and means or medians (standard deviations or ranges) for continuous and ordinal variables, respectively. The one‐way ANOVA was used to compare the differences between the groups. A p value less than 0.05 was considered significant.

## Results

### Video selection process

**Fig. 1:**
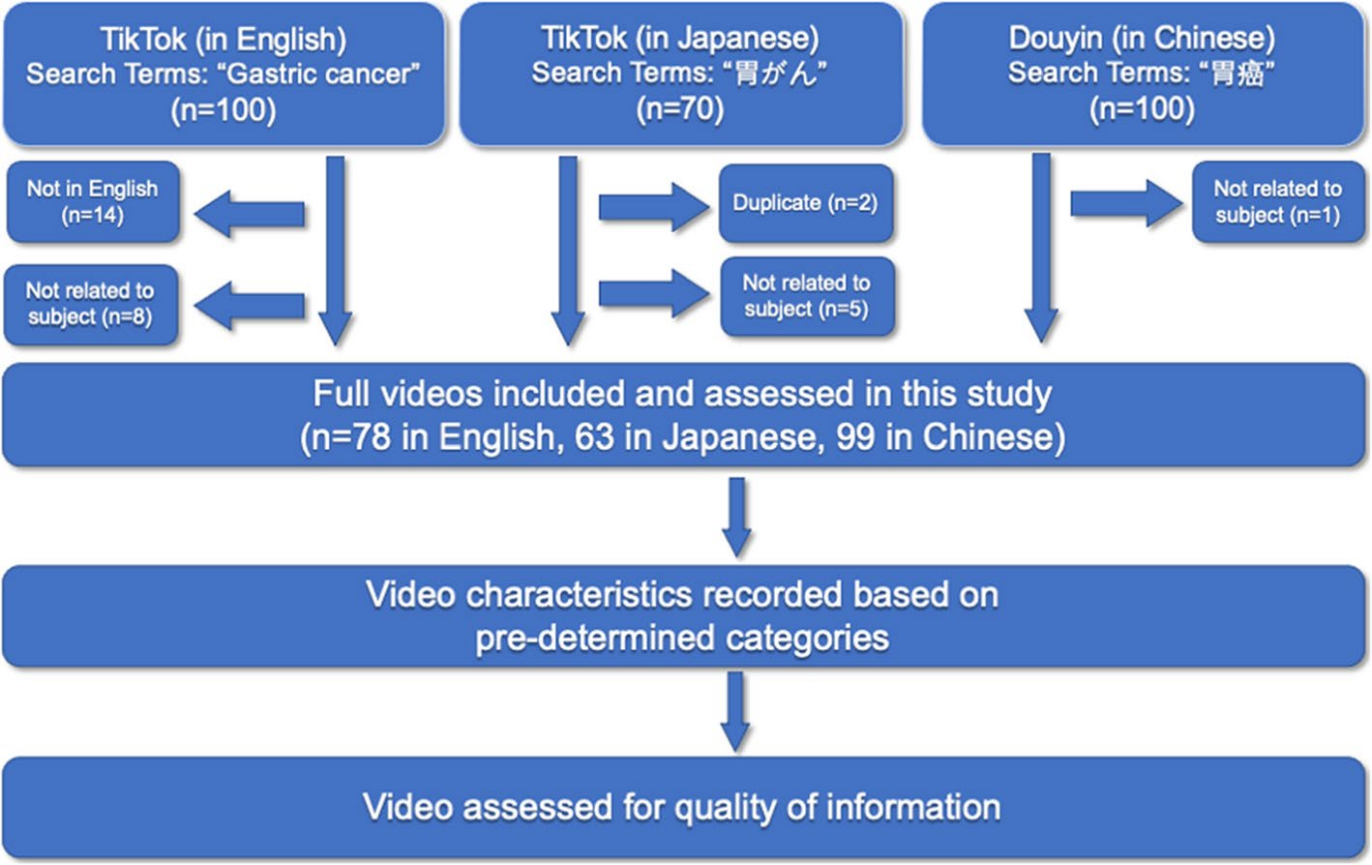
Flow diagram of video selection

More than 100 videos were presented in the results using English and Chinese keywords. However, only 70 videos were presented in the search result after inputting the Japanese keywords. Finally, we retrieved a total of 270 videos in the three languages. Of the 270 videos screened, 240 videos met the inclusion criteria. The video selection and analysis process are shown in Fig. 1.

### Video characteristics

The characteristics of the included videos are shown in Table 2. The mean length of duration for the videos was 30 s (9–80 s) in English, 35 s (9–60 s) in Japanese and 51 s (9–405 s) in Chinese. The mean video age was 134 days (5 to 528 days) in English, 180 days (11 to 1079 days) in Japanese, and 101 days (1 to 727 days) in Chinese. A total of 93.6% of the videos were in English and 95.2% in Japanese; they were uploaded on TikTok by private users. However, only 3% of private Chinese users uploaded their videos. Health professionals contributed the most videos in Chinese, accounting for approximately 57.6%. Among the useful videos, the videos published in Chinese had the highest QUEST (p < 0.05) and DISCERN scores (*p* < 0.05), followed by those published in Japanese.

**Table 2:** Characters of included videos

Category	Description	TikTok (In English) (*n* = 78)	TikTok (In Japanese) (*n* = 63)	Douyin (In Chinese) (*n* = 99)
Video source	Health professionals	5 (6.4%)	3 (4.8%)	57 (57.6%)
	Private users	73 (93.6%)	60 (95.2%)	3 (3.0%)
	News network	-	-	32 (32.3%)
	Others	-	-	7 (7.1%)
Video characteristics	Number of days online	134 (5–528)	180 (11–1079)	101 (1–727)
	Number of views	6597 (14–12.3 M)	-	-
	Number of likes	237 (0–2.9 M)	83 (0–41,700)	3568 (7–610.4 K)
	Number of comments	19 (0–22.5 K)	10 (0–1672)	118 (1-14 K)
	Video duration	30 (9–80)	35 (9–60)	51 (9–405)
Content	Patient experience/testimony	44 (56.4%)	26 (41.3%)	-
	Education	16 (21.8%)	11 (17.4%)	93 (93.9%)
	Patient support	16 (21.8%)	26 (41.3%)	6 (6.1%)
Information reliability	Useful	16 (20.5%)	11 (17.5%)	93 (93.9%)
	Useless	62 (79.5%)	52 (82.5%)	6 (6.1%)

Data are expressed as the number of cases (percentage) or median(range)

### Information reliability

The 270 included videos were categorized as useful and useless according to educational content (Table 2). The percentage of videos containing useful information was 20.5% in English, 17.5% in Japanese, and 93.9% in Chinese. Many videos were amateur videos about personal experience/testimony (56.4% in English and 41.3% in Japanese).

### Educational content

Useful videos were analyzed based on the educational information they contained (Table 3). In all the categories, the most frequently covered topic was prognosis (37.5%) in English, symptoms (54.5%) in Japanese, and preventative measures (47.3%) in Chinese. Table 4 shows the information completeness scores. The videos published by the health professionals had the highest total QUEST (*p* < 0.05) and DISCERN (*p* < 0.05) scores in Chinese. Videos by health professionals were significantly more complete than those posted by private users in all languages.

**Table 3:** Characteristics of educational videos

Variables	Tiktok In English			Tiktok In Japanese			Douyin In Chinese		
	Number of videos	Comments	Likes	Number of videos	Comments	Likes	Number of videos	Comments	Likes
Total	16	10,159	603,076	11	138	1379	93	63,722	1,878,772
Aetiology	3 (18.8%)	129 (1.3%)	1254 (0.2%)	1 (9.1%)	-	14 (1.0%)	39 (42.0%)	35,530 (55.8%)	1,425,509 (75.9%)
Anatomy	-	-	-	1 (9.1%)	-	5 (0.4%)	9 (9.7%)	1908 (3.0%)	73,547 (3.9%)
Symptoms	4 (25.0%)	205 (2.0%)	10,647 (1.8%)	6 (54.5%)	98 (71.0%)	1160 (84.1%)	38 (40.9%)	22,908 (35.9%)	438,435 (23.3%)
Preventions	3 (18.8%)	9725 (95.7%)	589,754 (97.8%)	-	-	-	44 (47.3%)	47,051 (73.8%)	1,310,276 (69.7%)
Treatments	3 (18.8%)	61 (0.6%)	863 (0.1%)	3 (27.3%)	40 (29.0%)	203 (14.7%)	5 (5.4%)	10,388 (16.3%)	88,120 (4.7%)
Prognosis	6 (37.5%)	111 (1.1%)	1511 (0.3%)	1 (9.1%)	-	11 (0.8%)	11 (11.8%)	1861 (2.9%)	31,882 (1.7%)

This percentage refers to the number of views out of the total number of comments or likes

**Table 4:** Completeness score of educational videos

Variables	TikTok (In English)	QUEST	DISCERN	Completeness score	TikTok (In Japanese)	QUEST	DISCERN	Completeness score	Douyin (In Chinese)	QUEST	DISCERN	Completeness score
Source of educational videos	16	10.80 ± 2.48	30.87 ± 3.70	1.18 ± 0.39	11	12.38 ± 1.50	31.44 ± 4.25	1.09 ± 0.29	93	13.10 ± 3.15	37.45 ± 4.90	1.65 ± 0.85
Health professionals	4 (25.0%)	11.50 ± 3.00	32.75 ± 5.68	1.50 ± 0.50	2 (18.2%)	13.50 ± 0.70	31.50 ± 0.71	1.50 ± 0.50	55 (59.1%)	13.88 ± 2.92	36.98 ± 4.05	1.65 ± 0.88
Private users	12 (75.0%)	10.640 ± 2.56	30.18 ± 2.75	1.08 ± 0.28	9 (81.8%)	12.00 ± 1.55	30.71 ± 4.86	1.00 ± 0.00	3 (3.3%)	10.50 ± 2.12	35.67 ± 2.52	1.00 ± 0.00
News network	-			-	-			-	28 (30.1%)	11.34 ± 2.96	35.10 ± 4.12	1.61 ± 0.77
Others	-			-	-			-	7 (7.5%)	9.80 ± 4.55	34.00 ± 5.89	2.00 ± 0.93

This percentage refers to the number of views out of the total number of educational videos

## Discussion

The use of video broadcasting sites as a source of information about malignant tumors, such as colorectal cancer [18], thyroid cancer [19], larynx cancer [20] and skin cancer [21], has been evaluated. Many studies have reported that video broadcast sites have positive and negative effects on health information dissemination. Some videos can provide useful information for surgery education [22, 23]. Videos may also promote misleading information, such as promoting -anorexia as a healthy lifestyle [24] and describing ineffective or potentially dangerous natural therapies for gallstone disease [25]. Not only were audience members attempting therapies that might be harmful, but they were not going in for proven therapies, which could lead to other complications.

TikTok, known in China as Douyin (literally “shaking sound” in Chinese), is a video-sharing platform and social networking service. TikTok and Douyin have almost the same user interface but no access to each other’s content. Since its launch in 2016, TikTok/Douyin has rapidly gained popularity worldwide.

There are huge differences in the global distribution of morbidity and mortality associated with gastric cancer. Half of the incidence of gastric cancer has been reported in East Asian countries, especially in Japan and China. The mortality from gastric cancer in Japan and China 'ranks first among all cancers. Japan’s is leading the way in the prevention, diagnosis and treatment of gastric cancer worldwide, which is considered a good example for Chinese doctors to follow. There are some differences in gastric cancer between the two countries. The 5-year survival of gastric cancer in China is low because more than 80% of patients are diagnosed at an advanced stage [26]. However, due to the national program for gastric cancer screening, the rate of diagnosis and treatment of early gastric cancer in Japan is 70% [27]. In this study, we also want to explore the difference between the contents in the two countries. The Japanese and Chinese keywords were searched in the video platforms.

Private users represent the greatest number of sources in English and Japanese. The contents were mainly about personal experiences regarding surgical procedures or hospital stays. Content concerning self-motivation or supportive families was found in some English and Japanese videos but not in Chinese videos. Patients in these developed countries were more optimistic than those in China. The Chinese videos uploaded by private users accounted for only 3%. Chinese cancer patients have a higher risk of anxiety and depression with cancer [28]. More than half of the patients in China did not know their exact diagnosis before chemotherapy [29]. In China, when disclosing a life-threatening diagnosis such as cancer to a patient, the “family consent for disclosure” approach is adopted by physicians [30]. Our results also show that English-speaking and Japanese doctors contribute fewer videos to the platforms. Some studies reported the use of other video-based platforms (e.g., Vimeo and YouTube) for medical education [31, 32]. Doctors in the United States and Japan may use such social media platforms to disseminate health information [33, 34]. They frequently experience intense and stressful work [35, 36] and tend to experience burnout, which may be the other reason for the result. Health professionals contributed the most videos in Chinese. Chinese patients prefer specialist hospitals and large hospitals, as measured by the number of beds and surgeries [37]. With the increasing popularity of Web 2.0 technologies, people are seeking health information online more frequently. Both the effort and reputation of physicians online contribute to the increased number of patient consultations [38]. To attract more patients, Chinese doctors are focusing on social media to extend their influence.

When video content was analyzed, the most popular video topics were prognoses in English, symptoms in Japanese, and preventative measures in Chinese. This may indicate that prognosis was the most important aspect for most English-speaking uploaders. The incidence of gastric cancer is steadily declining in Europe and the United States, and the overall five-year survival rate is 31% [39]. Most uploaders were private users who were more concerned about the prognosis. The aspect of symptoms is the most covered topic among Japanese individuals, which may be related to early cancer screening and the popularization of disease knowledge among the public in Japan. China has a great burden of gastric cancer, and the diagnosis rate of early-stage disease is relatively low. Awareness of the disease is essential for screening and early detection. The aspect of gastric cancer prevention has garnered increasing attention recently in China.

The average scores for completeness in our study were not high. Most of the analyzed videos only included one or two categories. It has been reported that videos with longer durations and higher video power indices seem to be associated with higher quality scores [19, 40]. Videos on TikTok are limited to 3 min. Due to the short length of videos, it is impossible to expect each video to comprehensively cover all aspects of gastric cancer; therefore, individuals will view videos that do not contain important and valuable content. However, recent research suggests that TikTok has great potential in conveying important public health messages to various segments of the population. Some videos can provide useful resources for information dissemination for chronic disease management [40] and personal protection [41]. Our results indicated that videos from health professionals have significantly higher completeness scores than those posted by private users. Studies on other video platforms, such as YouTube, also showed that the overall quality of the cancer videos was poor [42]. Health care professionals should be encouraged to upload cancer-related videos with accurate information to encourage patients to screen and direct them appropriately [43, 44]. Patients are increasingly turning to social media for health information, where most TikTok videos are posted by laypeople. The study illustrates that professionals should contribute more high-quality videos and leverage the power of this social media channel as a public information source.

There are limitations to our study. First, We only use QUEST and DISCERN to assess the quality of included videos. However, we encourage more studies using a variety of instruments to triangulate the validity of these findings in the future. Second we only used “gastric cancer” without including “stomach cancer” or “stomach neoplasm” to search videos on TikTok which may led miss some appropriate videos. Third, we only selected TikTok to analyze gastric cancer videos, given that it is reported as the most popular social media app. There are other platforms such as YouTube and Vimeo, etc. And content in other platforms may show different results and conclusions. Fourth, This study only comprises a snapshot of information when the study data were collected and may change due to new videos being uploaded or removed with time.

## Conclusions

Despite these limitations and delimitations, this study is one of the first to describe how TikTok is being used to disseminate information about gastric cancer. TikTok has great potential in conveying important health messages to public due to its widespread reach, but its limitations are also obvious, including too short videos containing not enough information, issues with unchecked spread of misinformation, difficulty identifying source credibility. TikTok in English and Japanese might not fully meet the gastric cancer information needs of public, but Douyin in Chinese was the opposite. Patients should remain cautious and selective when watching gastric cancer videos on TikTok. If patients want to seek useful information, it is better to seek videos uploaded by health professionals. It is necessary that healthcare professionals and academic institutions apply the characteristics of highly viewed video and think useful methods to solve the variable quality of information uploaded on TikTok. Content creators should be encouraged to direct public to evidence-based resources from health professionals and institutions. To maximize the potential of video-based information and minimize the quantity misleading or unhelpful information, multilateral efforts between healthcare professors, governments and social media platforms are needed.

## Data Availability

The datasets used and/or analysed during the current study available from the corresponding author on reasonable request.
